# Supplementary material for: Novel Driver Strength Index highlights important cancer genes in TCGA PanCanAtlas patients
Source: PeerJ. 2022 Aug 11;10:e13860. doi: 10.7717/peerj.13860 (PMC9375969; doi:10.7717/peerj.13860)
Supplement: Supplemental Information 2 [file peerj-10-13860-s002.zip › COHORTS/genes plots/age/2021_8_16_14_9_distribution_cohorts_males_50-54.pdf]

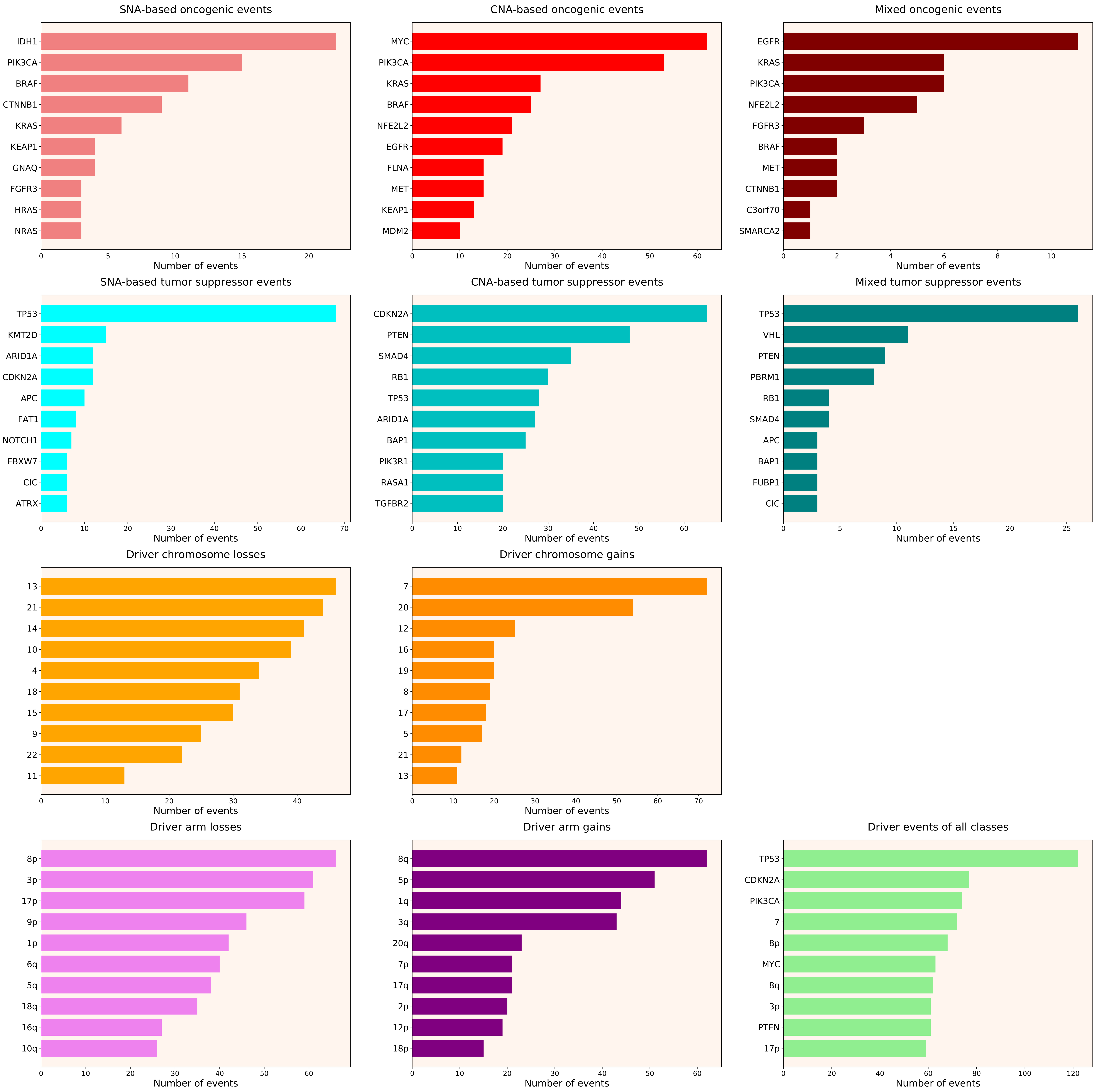

SNA-based oncogenic events
IDH1
PIK3CA
BRAF
CTNNB1
KRAS
KEAP1
GNAQ
FGFR3
HRAS
NRAS
Number of events
CNA-based oncogenic events
MYC
PIK3CA
KRAS
BRAF
NFE2L2
EGFR
FLNA
MET
KEAP1
MDM2
Number of events
Mixed oncogenic events
EGFR
KRAS
PIK3CA
NFE2L2
FGFR3
BRAF
MET
CTNNB1
C3orf70
SMARCA2
Number of events
SNA-based tumor suppressor events
TP53
KMT2D
ARID1A
CDKN2A
APC
FAT1
NOTCH1
FBXW7
CIC
ATRX
Number of events
CNA-based tumor suppressor events
CDKN2A
PTEN
SMAD4
RB1
TP53
ARID1A
BAP1
PIK3R1
RASA1
TGFBR2
Number of events
Mixed tumor suppressor events
TP53
VHL
PTEN
PBRM1
RB1
SMAD4
APC
BAP1
FUBP1
CIC
Number of events
Driver chromosome losses
13
21
14
10
4
18
15
9
22
11
Number of events
Driver chromosome gains
7
20
12
16
19
8
17
5
21
13
Number of events
Driver arm losses
8p
3p
17p
9p
1p
6q
5q
18q
16q
10q
Number of events
Driver arm gains
8q
5p
1q
3q
20q
7p
17q
2p
12p
18p
Number of events
Driver events of all classes
TP53
CDKN2A
PIK3CA
7
8p
MYC
8q
3p
PTEN
17p
Number of events
